# Clinical features, diagnostic findings, and treatment response in Finnish horses examined for equine inflammatory bowel disease

**DOI:** 10.1186/s13028-025-00831-8

**Published:** 2025-12-03

**Authors:** Henna Marjaana Pekkarinen, Umberto Simola, Kati Elina Niinistö, Pernilla Elisabet Sofia Syrjä

**Affiliations:** 1 Production and Companion Animal Pathology, Animal Health Diagnostic Unit, Finnish Food Authority, Mustialankatu 3, 00790 Helsinki, Finland; 2 Section for Pathology and Parasitology, Faculty of Veterinary Medicine, University of Helsinki, P.O. Box 57, Helsinki, Finland; 3 BC Platforms, Tykistökatu 4, 20520 Turku, Finland; 4 Veterinary Teaching Hospital, Equine Hospital, Faculty of Veterinary Medicine,, University of Helsinki, P.O. Box 57, Helsinki, Finland

**Keywords:** Equine IBD, Rectal biopsy, Treatment response

## Abstract

**Background:**

Equine inflammatory bowel disease (IBD) is challenging to diagnose and treat. Although the number of horses examined due to suspicion of IBD is increasing, the different treatments in clinical patients and their responses are not well documented. We sought to characterize the demography, signs, clinical and rectal biopsy findings, and treatment response in Finnish horses suspected to have IBD. Horses undergoing clinical examination due to suspected IBD in 2022 and with a good-quality rectal biopsy were selected for the study. General information, signs, clinical and histological findings, and treatment response were collected retrospectively from owners and participating clinics. The effect of variables on symptoms, treatment response, and biopsy results was assessed using statistical methods (significance level *P* < 0.05).

**Results:**

A total of 152 horses was included. The most common signs were poor performance (68%), nonspecific pain (43%), and irritation/aggression (41%). Intestinal signs were observed in 63% of horses. Sixty-six percent of horses had ultrasonographic changes in the small intestine, and 37% had gastric ulcers. Orthopaedic examination was mentioned in 12% of horses. In rectal biopsy, horses had eosinophilic (35%), lymphoplasmacytic (18%), or neutrophilic (6%) inflammation; 40% had no inflammation. Inflammation was observed mostly in horses aged 5–8 years (*P* = 0.015). Changes in rectal biopsy were associated with certain behaviour changes (*P* = 0.002). Sixty-eight percent of horses were treated with medication and dietary change. Glucocorticoids were first-line treatment in 73% of medicated horses. Medication was changed during treatment in 35 horses. Treatment response was considered good in 49% of horses. Access to pasture was associated with a reduction in signs (*P* = 0.001). Signs, clinical and biopsy findings, and treatment type were not associated with treatment response. Medication change was associated with poorer treatment response.

**Conclusions:**

Most horses had at least a partial positive treatment response. Investment in pasture-like management, forage, and exercise regimen may be helpful during out-of-pasture season. Performance issues and nonspecific behaviour changes should be included as possible IBD-related signs, but a more structured clinical diagnostic workup is needed for a reliable assessment of treatment response and to increase the diagnostic value of rectal biopsy.

**Supplementary Information:**

The online version contains supplementary material available at 10.1186/s13028-025-00831-8.

## Background

Equine inflammatory bowel disease (IBD) is a collective term for intestinal disorders with inflammation in the small or large intestine, or both [1, 2]. IBD remains poorly understood despite recent research [3, 4]. Equine IBD is often a diagnosis of exclusion and is characterized by nonspecific clinical signs, such as diarrhoea, unspecific pain, and irritation/aggression. The results of diagnostic tests can be highly variable [1], and there is an inconsistent response to treatment [3].

Common clinical signs of equine IBD are weight loss, chronic diarrhoea, and recurrent colic [1, 2], although lethargy can be the main sign [5]. It is not possible to obtain a definitive diagnosis of IBD in a live horse without biopsies obtained in hand-assisted laparoscopy or laparotomy. Although abnormal glucose absorption, hypoalbuminemia, hypoproteinaemia, and a thickened intestinal wall in abdominal ultrasonography are common findings in horses with IBD [1], these findings are not unique to IBD.

Intestinal biopsies remain the most popular diagnostic method used in horses to evaluate the inflammatory status of the intestine in suspected IBD [5]. The interpretation of equine intestinal biopsies (especially rectal biopsies) is unclear, especially when compared with small animals, where histopathological evaluation of intestinal disease is standardized [6]. Although some studies have characterized the normal quantity of intestinal inflammatory cells [7, 8], the rectal segment is rarely included. Preliminary studies of the inflammatory cell count in the duodenum and rectum of healthy horses indicate a higher cellularity in the small intestine [9]. Absolute cell numbers regarding cell type and numbers in the equine rectum have not been published. Therefore, the normal variation of inflammatory cells in the rectal mucosa and how well changes herein correlate with mucosal changes in other segments of the intestine remain unknown [1]. A Swedish study [10] presented the most comprehensive study on rectal biopsy diagnostics and proposed the broad classification of rectal biopsy findings currently available. Despite this, more recent equine IBD studies report histopathological changes in 38% [11] to 84% [5] of cases, indicating that there is still discrepancy in the definition of IBD and selection of cases. The inclusion criteria in recent studies varied from accepting horses solely with a clinical suspicion of IBD and an oral glucose absorption result [5] to requiring one clinical chemistry change or history of recurrent colic or weight loss with changes suggestive of IBD in rectal histopathology [11]. This further highlights the difficulties of diagnosing equine IBD, as a standardized classification system for histopathological interpretation is lacking and the clinical definition is inconsistent.

Based on intestinal histopathology, equine IBD can be divided into lymphoplasmacytic, eosinophilic, and granulomatous inflammation [1]. Although lymphoplasmacytic enterocolitis is rare in horses [12], its incidence is increasing [1]. One study [13] reported poor treatment response in lymphoplasmacytic IBD, but only four horses were treated with corticosteroids and cases with mild intestinal inflammation were excluded.

The prognosis of eosinophilic IBD depends on the underlying disease; the prognosis is poor when reported as part of a multisystemic eosinophilic epitheliotropic disease (MEED) [14]. In contrast, in intestine-limited eosinophilic enterocolitis (EC), the prognosis is better than in other forms of equine IBD [12]. Although granulomatous inflammation is considered to have the poorest prognosis [12], recent studies suggest that its incidence has markedly decreased [1]. Statistical analyses of the associations between histological inflammation type of the rectal biopsy and survival or treatment response of equine IBD are lacking in some of the most recent clinical studies [5, 11].

There are no standardized, evidence-based treatment recommendations for equine IBD [1]. Many suggested recommendations are based on limited research or extrapolate human and small-animal treatment recommendations [1, 2]. Histopathological diagnosis of rectal biopsy is sometimes used for follow-up [14], which further highlights the need for a more systematic histological interpretation standard. Currently, a tapered course of corticosteroids is the mainstay of treatment [2, 15] and correlates with a moderate long-term survival rate if the initial treatment response is good [11]. In cases with poor response to corticosteroids or an elevated risk of associated adverse effects (mainly laminitis), other immunosuppressive medications are used, such as sulfasalazine or azathioprine. There are limited studies on sulfasalazine and azathioprine in horses [1, 2, 16]. Dietary changes, such as an elimination diet or increasing the fibre content of feed, are often recommended for equine IBD [2, 15]. However, these approaches have not been shown to lead to an improved response to medical treatment [5].

In Finland, equine veterinarians are submitting increasing numbers of rectal biopsies with suspicion of IBD for histologic analysis. Currently, the main owner complaints have been poor performance or behaviour changes instead of gastrointestinal signs. Simultaneously, there is lack of knowledge on the diagnostic, prognostic, and predictive value of rectal biopsy findings in IBD. We sought to characterize the demography, clinical signs and findings, diagnostic workup, rectal biopsy findings, and treatment response in Finnish horses with suspected IBD.

## Methods

### Study population and inclusion criteria

Horses that had a rectal biopsy sample sent for histopathological evaluation either to a private veterinary pathology service (FACIT) owned by one of the authors (PS) or to the Faculty of Veterinary Medicine Section for Pathology and Parasitology between January 2022 and December 2022 were eligible for the study. Further inclusion criteria were that the rectal biopsy included the submucosa and an intact cross-section of the rectal mucosa, and that the clinical data were retrievable. Horses were examined at the University of Helsinki Equine Hospital, Hyvinkää Equine Hospital, Oulu Equine Clinic, or Equine Clinic Laukaa and Kuopio. The study was approved by the Research Ethics Committee on Animal Research of the University of Helsinki. Horses were included in the study only with informed consent from the owner.

### Histopathology

All biopsy reports were reviewed by the author HP, and information on histological findings, inflammatory type, and inflammation severity were recorded.

Biopsies from FACIT were analysed by one author (PS). The biopsies from the Faculty of Veterinary Medicine Pathology Department were originally analysed by various pathologists. Cases where the biopsy report did not align with the grading system outlined below were re-evaluated by PS.

**Fig. 1:**
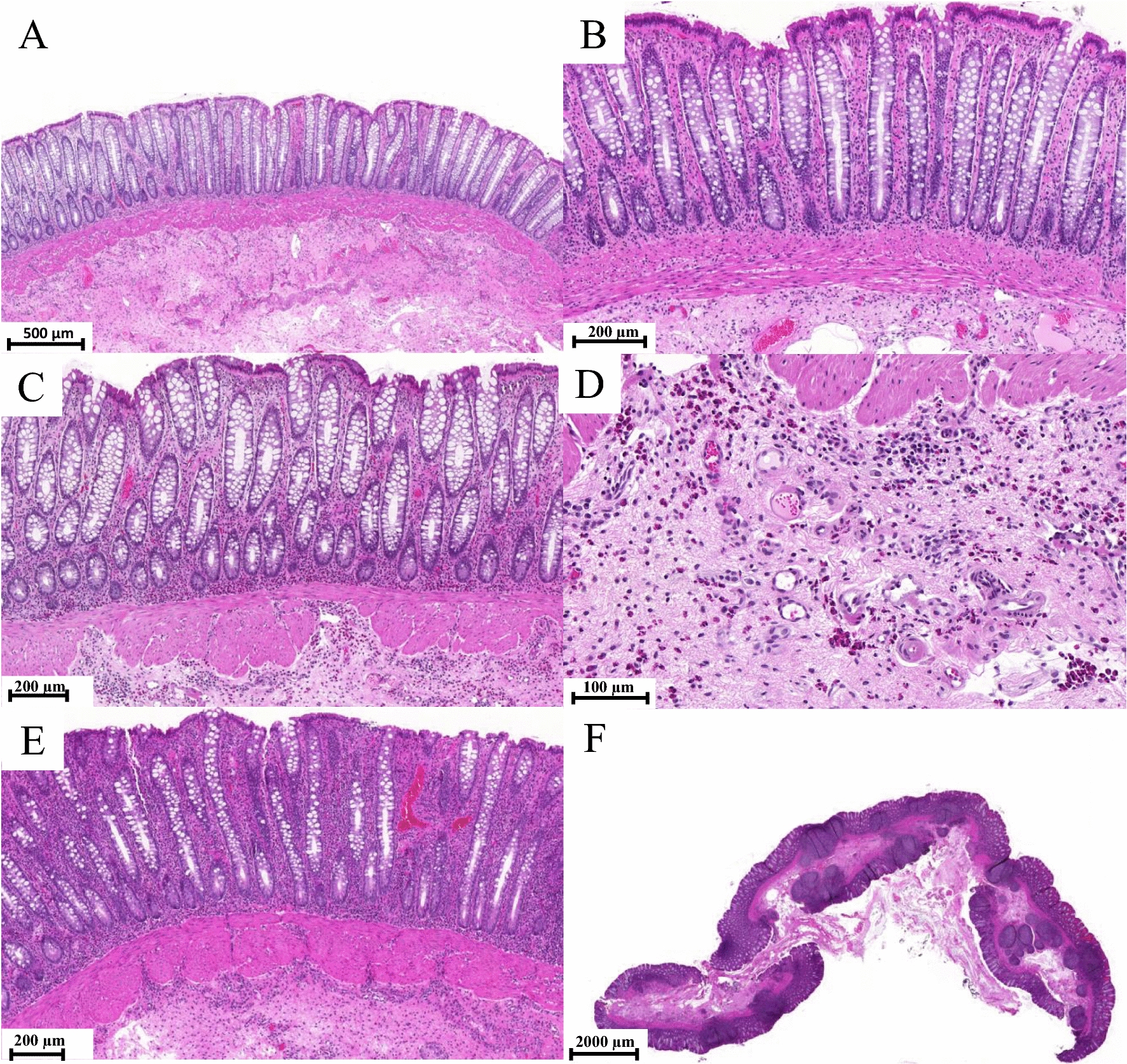
Outline for the categorization of inflammatory lesions in equine rectal mucosa. All samples were stained with HE. **A**, **B** Normal rectal mucosa. Epithelium is intact, the crypts are regularly arranged, and the cellularity of the propria is low. **C** Moderate eosinophilic inflammation with increased cellularity and increased irregularity of crypt arrangement. **D** Closer view of rectal submucosa showing clustering of eosinophils, which was used to detect eosinophilic inflammation in milder lesions. **E** Severe lymphoplasmacytic inflammation with increased cellularity and mild epithelial injury. **F** Lymphoid hyperplasia was interpreted as a finding supporting chronic inflammation in the gut

The rectal biopsies were histologically evaluated largely in line with previous reports and grading of equine rectal biopsies [10], aiming at an approach practical in routine diagnostic use. Cellularity was evaluated by the number of specific cells in a high-power field (HPF), which in a light microscope at 400 × magnification with an ocular FN of 22 was equal to 0.237mm^2^. Biopsies were classified as normal (Fig. 1 A and B) if the mucosal surface epithelium was intact, the crypts regularly arranged, and the proprial cellularity low, consisting mainly of lymphocytes and plasma cells (approximately 2–3 between neighbouring crypts). Low numbers of eosinophilic granulocytes (< 10 cells/HPF), mainly beneath the crypt basis in the propria and as single cells diffusely in the submucosa, were also considered normal. Neutrophilic granulocytes were not regularly detected in rectal biopsies that were classified as normal.

Eosinophilic inflammation (Fig. 1C) was graded as mild (< 20 cells/HPF), moderate (20–40 cells/HPF), or severe (> 40 cells/HPF). In particular, the eosinophilic reaction of the submucosa (Fig. 1D), with clustering of cells in groups, was used to detect milder lesions.

Lymphoplasmacytic inflammation (Fig. 1E) was graded as mild (< 5 cells), moderate (< 10 cells), or severe (> 10 cells) based on the approximated number of mononuclear cells between neighbouring crypts. Lymphoid hyperplasia in the samples was interpreted as a finding supporting chronic inflammation in the gut (Fig. 1F).

### Collection of case data and the owner questionnaire

Data from each horse’s signalment and history, main clinical signs, results of clinical examinations, and medications and feed modifications (along with associated responses) were collected from owners and electronic patient records in 2023. Additionally, data on pasture time and free text answers on clinical signs, treatment, and treatment response were collected from owners by email or by the Research Electronic Data Capture (REDCap) tools hosted at the University of Helsinki [17, 18]. The questionnaire contained multiple choice questions, dichotomous questions, and free text options (Additional file 1). All answers were optional. Owners were given 4 months to answer the electronic questionnaire. The responses were exported to Excel (v. 2308, Microsoft Corp., USA) from the REDCap platform. All information was pseudonymized.

Breeds with more than 10 individuals were clustered as a specific breed. Other breeds were grouped based on general breed characteristics and usage. The group “other” was used for rare breeds to ensure anonymity.

### Statistical analysis

Statistical analyses were performed on BC Platforms using SAS® System for Windows, version 9.4 (SAS Institute Inc., Cary, NC, USA). The Mehta and Patel tests (an extension of the Fisher’s exact test for larger RxC tables) [19, 20] were performed using the R System for Windows, version 4.2.2 [21].

To evaluate treatment response in relation to rectal biopsy findings, a sample size of 28 per group (no inflammation, eosinophilic inflammation, or lymphoplasmacytic inflammation) was sought to achieve a power level of 0.9.

To evaluate the statistical significance of the association between variables, Pearson’s χ^2^ test was used for dichotomous and nominal categorical variables. Mehta and Patel was applied in cases where there were fewer than five instances. Point biserial correlation was used to evaluate the association between continuous and dichotomous variables. Cramer’s V was used to evaluate the strength of the significance. The statistical significance level was set at *P* < 0.05.

Additional simplified analyses were performed after initial statistical analyses with all groups and findings. Stallions and geldings were combined when analysing the association of sex in rectal biopsy findings. The treatment response was simplified into the following two groups twice: at first “good response” and “no response first but good later” against the other options, then “good response” against all the other options. Rectal inflammatory lesions were grouped into “no inflammation + mild inflammation” and “moderate + severe inflammation” to determine if this affected treatment response. Signs on pasture were simplified into three groups (any positive change, any negative change, no change or not known) to identify even minor associations with treatment response or pasture access.

## Results

### Study population, rectal biopsy findings, and disease outcome

A total of 293 rectal biopsies were examined during 2022, including 248 high-quality rectal biopsies. Of these, 96 were excluded due to incomplete data or abstained owner consent to use the horse’s medical records, leaving 152 total included biopsies. Of the 152 owners, 118 completed the questionnaire at least partly.

**Fig. 2:**
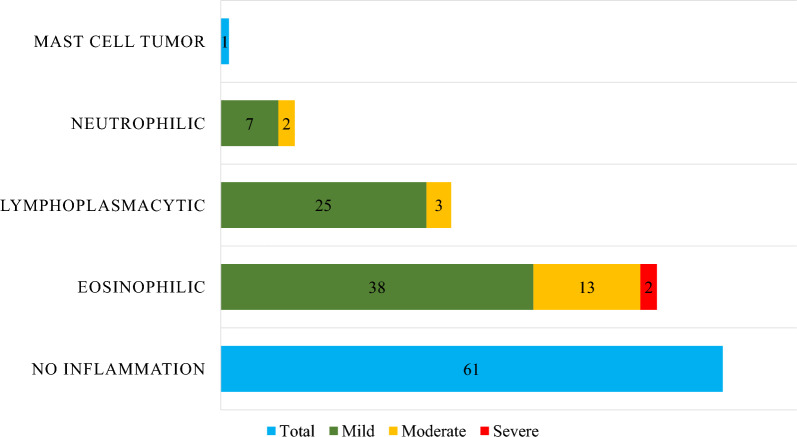
Distribution of rectal biopsy findings in all 152 horses, with colour-coded severity of inflammation. See text for the specification of grading. Numbers indicate the number of biopsies with each finding and its severity

The findings of the rectal biopsies are presented in Fig. 2. The most common diagnosis was no inflammation (40% of horses); 35% of the horses had eosinophilic, 18% lymphoplasmacytic, and 6% neutrophilic inflammation. Of the inflammatory changes, 78% were considered mild (70/90), 20% were considered moderate (18/90), and 2% were considered severe (2/90). One horse had changes indicative of a mast cell tumour and was not included in further statistical evaluation regarding the association between rectal findings and age, breed, sex, or signs on pasture.

Thirteen horses were reported to be deceased between the initial IBD examinations and the collection of data for this study. Three were euthanized because of unsatisfactory treatment response, two because of sudden general health deterioration, and one because of recurrent colic. Two horses were euthanized due to signs unrelated to IBD. One horse died suddenly, but no autopsy was performed. Cause of euthanasia was not given in four horses, in three of these the treatment response was initially good but deteriorated later.

### Associations between signalment, clinical signs, and rectal biopsy findings

The signalment of the horses is presented in Table 1. Inflammatory changes were seen most often in horses aged 5–8 years (χ^2^, *P* = 0.052) when analysing rectal biopsy changes within different age groups. Horses aged 9–12 years most commonly did not have any changes. There were no significant differences when comparing the rectal biopsy findings in each sex (χ^2^, *P* = 0.379) and breed group (χ^2^, *P* = 0.726).

**Table 1:** The main signalment of 152 horses

**Sex, number of horses per**^**a**^	
Mare	73
Gelding	74
Stallion	4
**Median age**^**b**^** (range), years**	8 (2 –20)
**Breed**^**c**^**, number of horses per**	
Warmblood riding horses other than Finnish warmblood (FWB)	41
Finnhorse	37
FWB	18
Ponies	16
Standardbred trotters	15
Cold-blood breeds other than Finnhorse	11
Other	8

^a^ Not provided for one horse

^b^ Not provided for three horses

^c^ Not provided for six horses

**Fig. 3:**
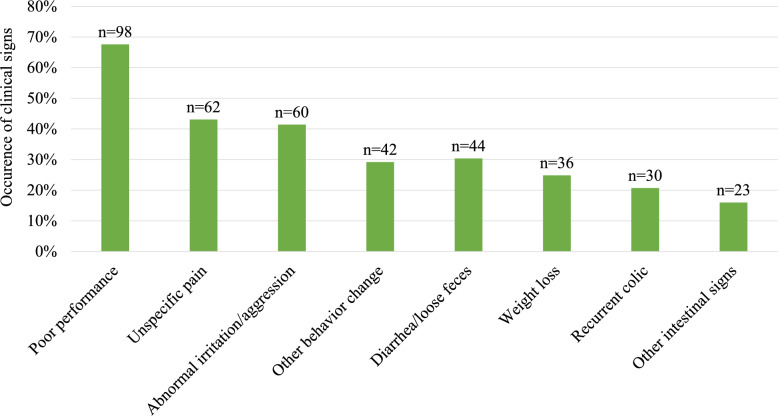
Main clinical signs of horses examined for inflammatory bowel disease. n = number of horses with sign in question. Other behavioural changes = abnormal stress behaviour, abnormal spooking, and self-isolation from group. Other gastrointestinal signs = free faecal water, gas accumulation, and frequent defecation

The main clinical signs are presented in Fig. 3. The most common clinical sign was poor performance, described either as performance issues or difficulties during riding. In the open-ended questions, 29 owners reported the horse protesting against the rider’s aids or refusing to move, becoming one-sidedly worse especially in canter, or starting to buck, rear, or both. Other behavioural changes included abnormal stress behaviour, abnormal spooking, and self-isolation from group; these changes were recorded more often in horses with rectal mucosal changes than in those with normal rectal biopsy (34/86 vs. 8/50, χ^2^, *P* = 0.002). Despite the low overall incidence of gastrointestinal signs, 63% of horses presented with at least one gastrointestinal sign. Other gastrointestinal signs included free faecal water, gas accumulation, and frequent defecation, with 7 horses having these as their only noted gastrointestinal sign. Statistically significant associations between the different types of rectal biopsy changes and the expressed signs were not detected for any of the considered categories. The expressed signs were also not significantly associated with treatment response for all considered categories, except for a weak trend between recurrent colic and a better treatment response (17/62 vs. 9/66, Mehta and Patel, *P* = 0.07; χ^2^, *P* = 0.086).

### Comparing subpopulations of horses with either poor performance or intestinal signs

To distinguish if rectal biopsy findings and treatment response were confounded by horses possibly not affected by IBD, we compared horses with poor performance as the main clinical complaint and without intestinal signs with horses with gastrointestinal (GIT) signs as the main clinical complaint with no history of performance issues. Data on rectal biopsy findings and treatment response in these subpopulations are presented in Additional file 2. Although rectal inflammation appeared to be more common in horses with poor performance without GIT signs than in horses with GIT signs, no statistically significant associations were seen between the subpopulations and rectal biopsy finding (χ^2^, *P* = 0.385), severity of rectal inflammation (χ^2^, *P* = 0.815), or treatment response (χ^2^, *P* = 0.145).

### Clinical examinations and findings in relation to rectal biopsy findings

**Fig. 4:**
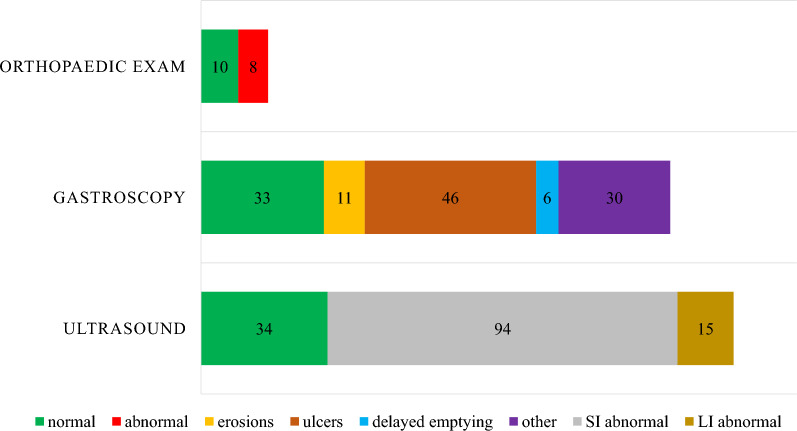
Selected clinical examinations performed on horses during examination for inflammatory bowel disease. SI = small intestine. LI = large intestine. Numbers indicate the number of horses with each clinical examination finding

Selected clinical examinations and their findings are presented in Fig. 4.

Abdominal ultrasonography was performed in 94% (143/152) of horses. Thickness of the small intestine wall was reported for 88 horses; the small intestine wall was considered abnormal (e.g. thickened or oedematous) for all of these horses. Median thickness was 4.45 mm (range 3–8 mm). Ultrasonographical changes had no association with rectum biopsy diagnosis (Point Biserial Correlation, *P* = 0.0976).

Gastroscopy was performed in 83% (126/152) of horses. There was no statistically significant association between gastric erosion or ulcer and clinical signs for any of the considered categories or treatment response (17/38 vs. 20/39, χ^2^, *p* = 0.729).

Orthopaedic examination was performed for 12% (18/152) of horses. Treatment response was recorded in 15 horses that underwent orthopaedic examination, of which 11 were treated for IBD, 3 had no treatment, and one was given antibiotics. Interestingly, a statistically significant association was seen between orthopaedic examination and a poorer treatment response (2/63 vs. 13/67, χ^2^ *P* = 0.009), despite the results of the orthopaedic examination not being clearly documented.

### Treatment choices

Treatment information was obtained from 92% (140/152) of horses. In 68% (95/140) of horses, the treatment was a combination of medication and dietary change. Only medication was given to 19% (26/140), and only dietary change to 10% (14/140) of horses, with 3.6% of horses (5/140) receiving no treatment. Dietary changes included replacing current hay with a more easily digestible type of hay, removing processed feed from the diet, and restricting feed to only dry hay and a mineral supplement, with oats in some cases. No significant association was detected between treatment type and treatment response in horses that had a reported result for both (n = 130, χ^2^, *P* = 0.839).

Information on medication was provided for 124 horses. As a first-choice medication, glucocorticoids were used in 73% (90/124) of horses and 6.5% (8/124) received sulfasalazine/azathioprine. In 3.2% (4/124) of horses, a combination of a glucocorticoid (prednisolone or dexamethasone) and either sulfasalazine or azathioprine was used. Non-IBD medications were given for 18% (22/124) of horses and included misoprostol, metronidazole, and metoclopramide. For first-choice medication, no significant association was seen between chosen medication and the treatment response (χ^2^, *P* = 0.141).

Medication was changed during treatment for 35 horses. In 51% (18/35) the medication was changed to azathioprine, and all but one of these horses were first given glucocorticoids. Other medications were used less, with 14% (5/35) changed to glucocorticoids, 11% (4/35) changed to sulfasalazine, and 5.7% (2/35) changed to metronidazole. In 17% (6/35) of horses, multiple medication changes were made. In horses with a change in medication, the overall treatment response was rarely considered good by the owner (8/57 vs. 23/64 horses, Mehta and Patel, *P* = 0.007; χ^2^, *P* = 0.011). However, the type of second medication had no significant association with treatment response (χ^2^, *P* = 0.156).

### Treatment response

**Fig. 5:**
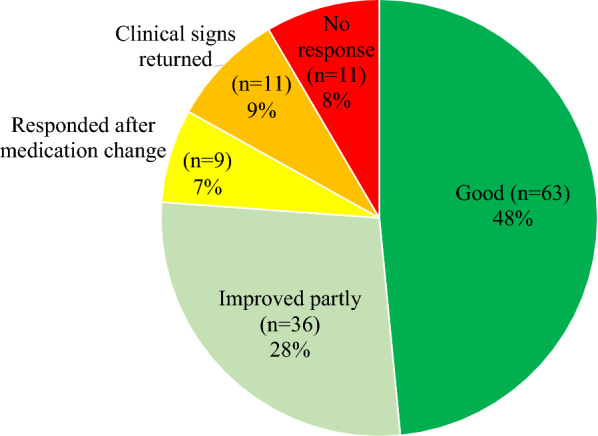
Distribution of the owner evaluation of treatment response in 130 horses. n = Number of horses with each treatment response evaluation

Owner’s assessment of the treatment response was provided for 86% (130/152) of horses. The treatment response was generally considered good, and most horses responded positively at least partly. The distribution of different treatment outcomes is presented in Fig. 5. There was no statistically significant association between treatment response and histological type (χ^2^, *P* = 0.699) or severity of the inflammatory lesion (11/66 vs. 7/63, χ^2^, *P* = 0.512).

### Effects of pasture on symptoms and rectal biopsy findings

Information about access to pasture was obtained from 74% (112/152) of horses, and information about how the signs changed on pasture was obtained from 74% (113/152) of horses. Half of the horses (56/112) were on pasture full time during pasture season (approximately May–August in Finland), with 31% (35/112) being part-time and 19% (21/112) having no access to pasture. The signs reduced in 53% (60/113) of horses and worsened in 6.2% (7/113). In 41% (46/113) of horses, the signs remained the same or it was not known whether the signs changed.

Access to pasture was significantly associated with a reduction in signs (χ^2^, *P* = 0.004). In horses that did not go to pasture, the signs remained the same or owners could not tell if they changed (Mehta and Patel, *P* = 0.001). There was no statistically significant association between daily pasture time and treatment response (χ^2^, *P* = 0.214) or between histopathological findings when comparing horses that expressed signs on pasture with those that did not (χ^2^, *P* = 0.422).

## Discussion

We sought to characterize the demography, signs, clinical findings, diagnostic workup, rectal biopsy findings, and treatment response in Finnish horses suspected to have IBD. Based on our study, Finnish horses responded well to treatment, and access to pasture significantly alleviated symptoms. However, we found that very few factors are linked to rectal biopsy diagnosis or a positive treatment response, and our findings do not support the usefulness of rectal biopsy histopathology in its current form for treatment selection. Our data highlight the need for a more structured approach in the diagnostics and treatment of equine IBD.

### The role of rectal biopsy

Neither clinical signs nor treatment response were associated with a certain inflammatory type in the rectum, thus the diagnostic value of the rectal biopsy is small in suspected IBD cases in Finland. Mild inflammatory changes were the most common histological findings in our study population. This may decrease the significance and complicate alignment of our findings with the current literature, since cases with mild rectal inflammation have been excluded in some previous studies [13]. However, it is unlikely that all our findings represent normal variation, as several rectal biopsies showed moderate or severe inflammatory changes and the percentage of rectal biopsies with no inflammatory changes corresponded with the current literature [5, 10].

Eosinophilic inflammation was the most common inflammatory change observed, which is consistent with previous studies [12]. The relatively high prevalence of lymphoplasmacytic inflammation is also consistent with a recent study [1]. Inflammatory changes were most common in relatively young horses, which have a more pronounced inflammatory response to any insult than older animals. However, the median age of the study cohort was also relatively low, implying that IBD-associated signs are expressed and investigated especially in young adult horses. Although functional gastrointestinal disorders (such as irritable bowel syndrome [IBS]) have not been described in horses, it is possible that horses have similar disorders [22]. An IBS-like syndrome in horses may explain the relatively high percentage of normal rectal biopsy findings observed both in our study and in the literature.

As it remains unknown how well the rectal mucosa mirrors inflammatory changes in other parts of the equine intestine, more studies on the normal variation of rectal inflammatory cell numbers are needed. We found no difference in the degree of inflammatory changes in the rectal biopsy of horses with only poor performance compared with horses with only gastrointestinal complaints. In fact, some horses with gastrointestinal signs had milder inflammatory lesions than those with only poor performance.

### Performance-related signs in suspected IBD cases

Poor performance and behaviour changes were observed in many of our suspected IBD cases. Extraintestinal signs are infrequently discussed in the equine IBD literature, although reduced performance and sensitivity to touch were noted in a recent publication [3]. Increased awareness of both veterinarian and owner to signs of pain or behaviour changes due to pain may lead to horses being examined sooner, even before clear intestinal signs arise. Extra-intestinal signs, such as itching and decreased activity, have also been reported in dogs with chronic enteropathy [23]. Other behaviour changes (abnormal stress behaviour, abnormal spooking, and self-isolation from group) were also significantly associated with changes in the rectal mucosa. Signs of pain are not always related to the organ of origin in horses; for example, dental pain can have various forms [24]. IBD or other gastrointestinal disorders should therefore be considered as a differential diagnosis even in the absence of intestinal signs, if other causes of pain (orthopaedic, dental) are excluded.

In our study, only a few horses with performance issues or riding difficulties underwent orthopaedic examination prior to rectal biopsy. Additionally, there was a negative correlation between orthopaedic examination and treatment response. The small sample size most likely caused at least moderate selection bias in this association. Non-gastrointestinal reasons may explain why these horses were more likely to respond unfavourably when treated for IBD. It is known that the initial response to IBD treatment mirrors long-term prognosis of the disease [11]. Therefore, a poor initial response in a horse with performance issues treated for IBD but without the exclusion of extraintestinal pain may even lead to a decision to euthanize the horse. This is why it is imperative to exclude other causes of poor performance and pain during clinical examinations before diagnosing IBD. Orthopaedic diagnostic and treatment regimens are currently better defined than those of equine IBD and could therefore decrease the number of suspected IBD patients undergoing rectal biopsy.

### Treatment response

The treatment response in our study was more favourable than the recently reported prognosis of fair to moderate [1]. Since there currently is no universally accepted clinical definition for IBD used in the inclusion criteria of studies, comparison of treatment responses between different studies is difficult, and the true clinical prognosis of IBD remains unclear. Since the owner performed most of the evaluation of treatment response in our study, this subjective evaluation probably caused bias. However, as the alleviation of signs is the most important goal of IBD treatment, we concluded that owner evaluation was a reasonable approach to gather sufficient data. Another limitation regarding the observed treatment response in this study is how many of the treated horses truly had IBD. Considering the variation in clinical workup of the horses, it is likely that our cohort included horses that were treated for IBD but in fact may have had other diseases. Further studies with narrower clinical inclusion criteria are needed to more accurately evaluate the treatment response of IBD.

The change of medication associated with a poorer treatment response is consistent with the findings of a previous study [11], as rapid response usually eliminates the need for treatment change. It is important to note that the small sample size likely caused at least moderate selection bias in our data. However, IBD medication is usually changed due to unsatisfactory initial response, and our study also had horses that benefitted from medication change. Therefore, available optional treatment options should be considered and may improve outcomes in some cases. Notably, the treatment response can also deteriorate with time, which should be communicated to the owner.

### Effect of pasture access on perceived signs

Giving the horse access to pasture reduced clinical signs when compared with horses that did not have access. This may not only be due to the change in forage but also to changes in the amount of exercise, feeding times, amount of feed, and type of feed when compared with indoor months. On pasture, horses walk slowly most of the day, eat very frequently, and eat fresh grass-based feed. This is very different from the typical indoor months in Finland, when horses are fed four to five times a day with dry hay or haylage and spend most of time in dirt paddocks or in the stable. Investigating management during out-of-pasture season and addressing forage type and exercise regimen may be helpful in controlling clinical signs.

### Other findings

Intestinal wall thickness has been evaluated in numerous studies, with normal values ranging from 2 to 4 mm [25–28]. A previous study [29] concluded that 5-mm diffuse thickness and 5.7-mm focal thickness were the best cut-off values for sensitivity and specificity for diagnosis of equine IBD. However, another study [5] found no association between thickened intestinal wall and abnormal duodenal biopsy. In our data, the upper limit for normal intestinal wall thickness in the ultrasonographical examination varied depending on the attending veterinarian. Furthermore, the exact intestinal wall thickness was only mentioned when considered abnormal. Due to these limitations, only comparison of an ultrasonographically detected change and histopathological diagnosis was analysed with nonsignificant results. As ultrasonography is non-invasive and feasible to perform and reproduce, it would be beneficial to have more studies on its usefulness in IBD diagnosis in horses.

Although gastric changes were not significantly associated with signs or treatment response in our study, it is important to note that more than one-third of horses had gastric ulcers at the time of examination. Gastroscopy is often mentioned as a part of the clinical procedures for diagnosis of IBD [1, 15], and gastric ulcer pain sometimes manifests with similar signs as IBD.

## Conclusions

We present the demographics, clinical signs, rectal biopsy findings, and treatment response of Finnish horses suspected of IBD. Our results indicate that performance issues are common clinical signs in horses examined for IBD, access to pasture alleviates clinical signs, rectal biopsy alone does not have a high predictive value, and treatment response is mostly good. Our findings are novel and relevant, as previous studies present data from more temperate climates and horses of different breed and housing than those of the Finnish horse population. This study provides a basis of information about the current diagnostic approach, treatment, and possible outcomes in equine IBD in Finland and highlights the multiple challenges that should be addressed in future studies.

## Supplementary Information

Additional file 1.

Additional file 2.

## Data Availability

The fully anonymized datasets used and analysed during the current study are available from the corresponding author on reasonable request.

## Declarations

### Prior publication

Parts of the data included in this article have previously been presented in the 2024 Finnish Veterinary Congress, Helsinki, Finland, 13–15 November 2024.

## References

[1] Vitale V. Inflammatory bowel diseases in horses: what do we know? Equine Vet Educ. 2022;34(9):493–500.

[2] Timko K. Approach to inflammatory bowel disease. Vet Clin North Am Equine Pract. 2024;40:287–306. 38789349 10.1016/j.cveq.2024.04.004

[3] Kranenburg LC, Bouwmeester BF, van den Boom R. Findings and prognosis in 149 horses with histological changes compatible with inflammatory bowel disease. Animals. 2024;14:1638. 10.3390/ani14111638. 38891685 10.3390/ani14111638 PMC11171156

[4] Sävilammi T, Alakangas R-R, Häyrynen T, Uusi-Heikkilä S. Gut microbiota profiling as a promising tool to detect equine inflammatory bowel disease (IBD). Animals. 2024;14:2396. 10.3390/ani14162396. 39199930 10.3390/ani14162396 PMC11350833

[5] Boshuizen B, Ploeg M, Dewulf J, Klooster S, de Bruijn M, Picavet M-T, et al. Inflammatory bowel disease (IBD) in horses: a retrospective study exploring the value of different diagnostic approaches. BMC Vet Res. 2018;14:21. 10.1186/s12917-018-1343-1. 29351774 10.1186/s12917-018-1343-1 PMC5775604

[6] Day MJ, Bilzer T, Mansell J, Wilcock B, Hall EJ, Jergens A, et al. Histopathological standards for the diagnosis of gastrointestinal inflammation in endoscopic biopsy samples from the dog and cat: a report from the World Small Animal Veterinary Association Gastrointestinal Standardization Group. J Comp Pathol. 2008;138:S1-43. 18336828 10.1016/j.jcpa.2008.01.001

[7] Packer M, Patterson-Kane JC, Smith KC, Durham AE. Quantification of immune cell populations in the lamina propria of equine jejunal biopsy specimens. J Comp Pathol. 2005;132:90–5. 15629483 10.1016/j.jcpa.2004.06.002

[8] Rocchigiani G, Ricci E, Navarro MA, Samol MA, Uzal FA. Leukocyte numbers and intestinal mucosal morphometrics in horses with no clinical intestinal disease. J Vet Diagn Invest. 2022;34:389–95. 34293980 10.1177/10406387211031944 PMC9254073

[9] Robel M, Grest P, Schoster A. Quantification of immune cell populations in equine intestinal biopsies of healthy horses. J Vet Intern Med. 2024;38:1246. 10.1111/jvim.17022.

[10] Lindberg R, Nygren A, Persson SG. Rectal biopsy diagnosis in horses with clinical signs of intestinal disorders: a retrospective study of 116 cases. Equine Vet J. 1996;28:275–84. 8818593 10.1111/j.2042-3306.1996.tb03091.x

[11] Kaikkonen R, Niinistö K, Sykes B, Anttila M, Sankari S, Raekallio M. Diagnostic evaluation and short-term outcome as indicators of long-term prognosis in horses with findings suggestive of inflammatory bowel disease treated with corticosteroids and anthelmintics. Acta Vet Scand. 2014;56:35. 10.1186/1751-0147-56-35. 24894126 10.1186/1751-0147-56-35 PMC4055252

[12] Schumacher J, Edwards JF, Cohen ND. Chronic idiopathic inflammatory bowel diseases of the horse. J Vet Intern Med. 2000;14:258–65. 10830538 10.1892/0891-6640(2000)014<0258:ciibdo>2.3.co;2

[13] Kemper DL, Perkins GA, Schumacher J, Edwards JF, Valentinen BA, Divers TJ, et al. Equine lymphocytic-plasmacytic enterocolitis: a retrospective study of 14 cases. Equine Vet J. 2010;32:108–12. 10.1111/j.2042-3306.2000.tb05346.x 11202375

[14] Kerbyson N, Knottenbelt D. Intestinal biopsies for investigating and managing inflammatory bowel disease in horses. In Pract. 2015;37:347–58.

[15] Kalck KA. Inflammatory bowel disease in horses. Vet Clin North Am Equine Pract. 2009;25:303–15. 19580941 10.1016/j.cveq.2009.04.008

[16] Valle E, Gandini M, Bergero D. Management of chronic diarrhea in an adult horse. J Equine Vet Sci. 2013;33:130–5.

[17] Harris PA, Taylor R, Thielke R, Payne J, Gonzalez N, Conde JG. Research electronic data capture (REDCap)—a metadata-driven methodology and workflow process for providing translational research informatics support. J Biomed Inform. 2009;42:377–81. 18929686 10.1016/j.jbi.2008.08.010 PMC2700030

[18] Harris PA, Taylor R, Minor BL, Elliott V, Fernandez M, O’Neal L, et al. The REDCap consortium: building an international community of software platform partners. J Biomed Inform. 2019;95:103208. 10.1016/j.jbi.2019.103208. 31078660 10.1016/j.jbi.2019.103208 PMC7254481

[19] Mehta CR, Patel NR. Algorithm 643: FEXACT: a FORTRAN subroutine for Fisher’s exact test on unordered r×c contingency tables. ACM Trans Math Softw. 1986;12:154–61.

[20] Mehta CR, Patel NR. A network algorithm for performing Fisher’s exact test in r × c contingency tables. J Am Stat Assoc. 1983;78:427–34.

[21] R Core Team. R: A language and environment for statistical computing. R Foundation for Statistical Computing, Vienna, Austria. https://www.R-project.org/ (2022). Accessed 9 Feb 2025.

[22] Hunter JO. Do horses suffer from irritable bowel syndrome? Equine Vet J. 2009;41:836–40. 20383978 10.2746/042516409x474284

[23] Holmberg J, Pelander L, Ljungvall I, Harlos C, Spillmann T, Häggström J. Chronic enteropathy in dogs – epidemiologic aspects and clinical characteristics of dogs presenting at two Swedish animal hospitals. Animals. 2022;12:1507. 10.3390/ani12121507. 35739843 10.3390/ani12121507 PMC9219460

[24] Pehkonen J, Karma L, Raekallio M. Behavioral signs associated with equine periapical infection in cheek teeth. J Equine Vet Sci. 2019;77:144–50. 31133309 10.1016/j.jevs.2019.03.005

[25] Beccati F, Pepe M, Gialletti R, Cercone M, Bazzica C, Nannarone S. Is there a statistical correlation between ultrasonographic findings and definitive diagnosis in horses with acute abdominal pain? Equine Vet J Suppl. 2011;98–105. 10.1111/j.2042-3306.2011.00428.x 21790762

[26] Bithell S, Habershon-Butcher JL, Bowen IM, Hallowell GD. Repeatability and reproducibility of transabdominal ultrasonographic intestinal wall thickness measurements in thoroughbred horses. Vet Radiol Ultrasound. 2010;51:647–51. 21158239 10.1111/j.1740-8261.2010.01715.x

[27] Epstein K, Short D, Parente E, Reef V, Southwood L. Gastrointestinal ultrasonography in normal adult ponies. Vet Radiol Ultrasound. 2008;49:282–6. 18546787 10.1111/j.1740-8261.2008.00367.x

[28] Kirberger RM, van den Berg JS, Gottschalk RD, Guthrie AJ. Duodenal ultrasonography in the normal adult horse. Vet Radiol Ultrasound. 1995;36:50–6.

[29] Ceriotti S, Zucca E, Stancari G, Conturba B, Stucchi L, Ferro E, et al. Sensitivity and specificity of ultrasonographic evaluation of small intestine wall thickness in the diagnosis of inflammatory bowel disease in horses: a retrospective study. J Equine Vet Sci. 2016;37:6–10.

