# Supplementary material for: Clinical features, diagnostic findings, and treatment response in Finnish horses examined for equine inflammatory bowel disease
Source: Acta Vet Scand. 2025 Dec 3;68:2. doi: 10.1186/s13028-025-00831-8 (PMC12781275; doi:10.1186/s13028-025-00831-8)
Supplement: Supplementary file 1 — Additional file 1. [file 13028_2025_831_MOESM1_ESM.pdf]

Additional file 1. Owner questionnaire

Owner questionnaire used to collect data on pasture time and free text answers on clinical signs, treatment, and treatment response. Translated from Finnish.

Horse's name is required to connect the questionnaire to the patient. All other questions are optional.

1. Name of the horse:
2. Geographical location:
   a. ☐ Southern Finland (South Karelia, Kanta-Häme, Kymenlaakso, Päijät-Häme, Uusimaa)
   b. ☐ Eastern Finland (South Savo, North Karelia, North Savo)
   c. ☐ Lapland
   d. ☐ South-Western Finland (Satakunta, Southwest Finland)
   e. ☐ Western and Central Finland (South Ostrobothnia, Central Ostrobothnia, Central Finland, Pirkanmaa, Ostrobothnia)
   f. ☐ Northern Finland (Kainuu, North Ostrobothnia)
   g. ☐ Åland
3. Year of birth: ____________
4. Sex:
   a. ☐ Mare
   b. ☐ Stallion
   c. ☐ Gelding
5. Breed: _____________
6. The primary use of the horse (choose the one that fits the best)
   a. ☐ Trotter
   b. ☐ Riding, hobby
   c. ☐ Riding, competitive
   d. ☐ Combined driving
   e. ☐ Companion horse (not exercised by human)
   f. ☐ Other
7. The primary use of the horse, if you wish to specify the answer "other:" ___________
8. Housing type
   a. ☐ Loose box
   b. ☐ Field shelter
   c. ☐ Activity-encouraging field shelter
   d. ☐ Mobile stable
   e. ☐ Other, what (please specify below)
9. Housing type, if you picked "Other what:" _____________
10. The horse is outside (in a paddock or field shelter) (if the horse is both alone and in group, pick the one that best describes the usual situation)
    a. ☐ Alone
    b. ☐ In group (one or more animal of the same species)
11. How long is the horse outdoors during barn feeding season? (pick the time that most closely fits a typical day or an average)
    a. ☐ Less than 4 h
    b. ☐ 4-8 h

   c. ☐ 8-12 h
   d. ☐ 12-16 h
   e. ☐ More than 16 h
12. How long is the horse outdoors during summer season? (pick the time that most closely fits a typical day or an average)
   a. ☐ Less than 4 h
   b. ☐ 4-8 h
   c. ☐ 8-12 h
   d. ☐ 12-16 h
   e. ☐ More than 16 h
13. The horse is on a green pasture during the summer
   a. ☐ Yes
   b. ☐ No
   c. ☐ Part-time (i.e. half a day or during the night)
14. The main signs of the horse before IBD examinations (choose the signs that were the reason why you contacted the veterinarian)
   a. ☐ Intermitted/continuous diarrhoea or looseness of faeces
   b. ☐ Recurrent colic
   c. ☐ Weight loss
   d. ☐ Performance issues or difficulties with riding (e.g. loss of speed, bucking)
   e. ☐ Irritation or aggression
   f. ☐ Unspecific signs of pain
   g. ☐ Other, what
15. You can specify signs here: _____________
16. Did the signs change during pasture season?
   a. ☐ Reduced by a lot
   b. ☐ Reduced a little
   c. ☐ Didn't change
   d. ☐ Worsened a little
   e. ☐ Worsened a lot
   f. ☐ I cannot say
17. Treatment of the horse after diagnosis (choose all that apply)
   a. ☐ Medical treatment (e.g. prednisolone, dexamethasone, salazopyrine, imurel)
   b. ☐ Change of feeding (e.g. Elimination diet, change of hay)
   c. ☐ No treatment
18. You can specify treatment here: ______________
19. Did the treatment help with the noted signs?
   a. ☐ Yes
   b. ☐ Partly
   c. ☐ No
20. You can specify the treatment response here: _____________
21. You can give more information about any question or give other additional information here: _______________
