# Supplementary material for: Clinical features, diagnostic findings, and treatment response in Finnish horses examined for equine inflammatory bowel disease
Source: Acta Vet Scand. 2025 Dec 3;68:2. doi: 10.1186/s13028-025-00831-8 (PMC12781275; doi:10.1186/s13028-025-00831-8)
Supplement: Supplementary file 2 — Additional file 2. [file 13028_2025_831_MOESM2_ESM.pdf]

Additional file 2. Distribution of rectal inflammation and treatment response of horses in subgroups "only performance issues, no intestinal signs," and "only intestinal signs, no performance issues."

A. Rectal inflammation within subgroups, total of 77 horses (42 in "only performance" and 35 in "only gastrointestinal").

B. Treatment response within subgroups, total of 64 horses (35 in "only performance" and 29 in "only gastrointestinal").

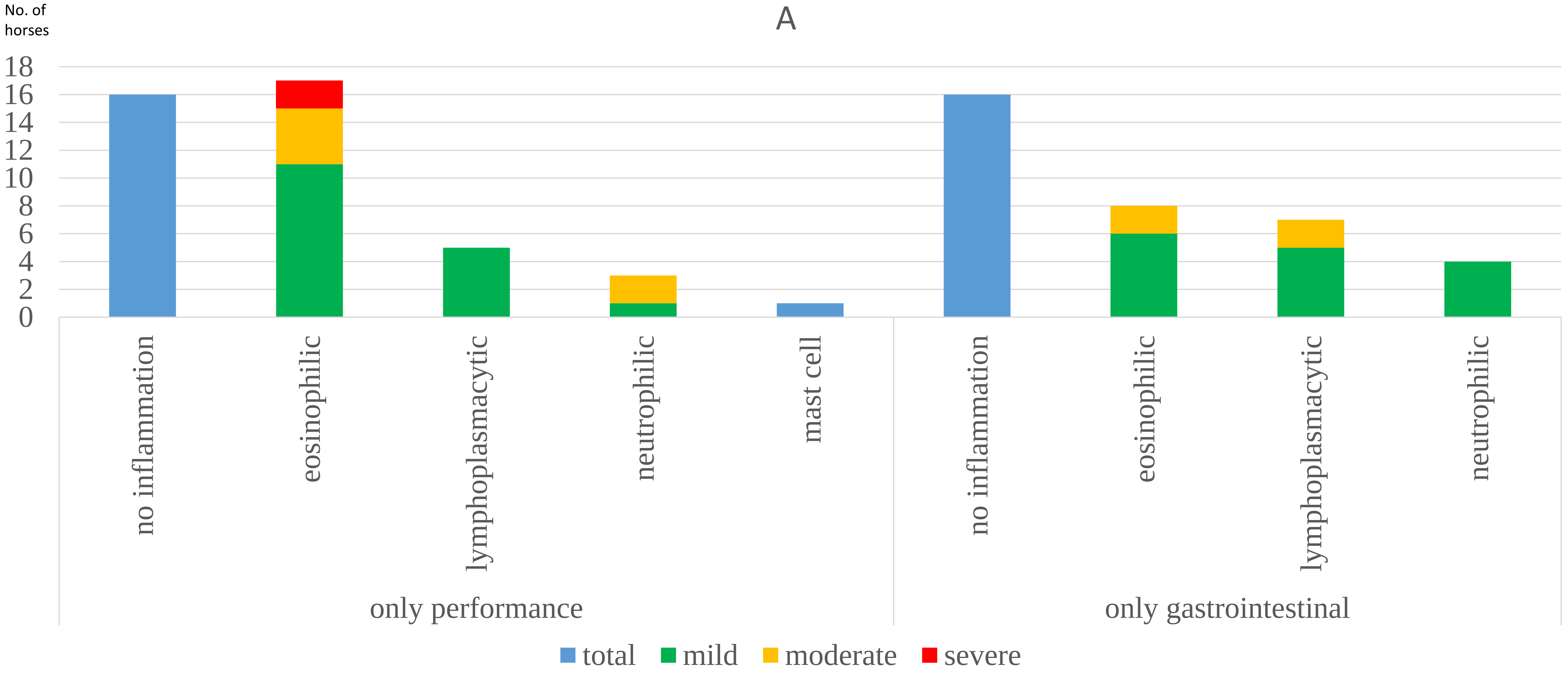

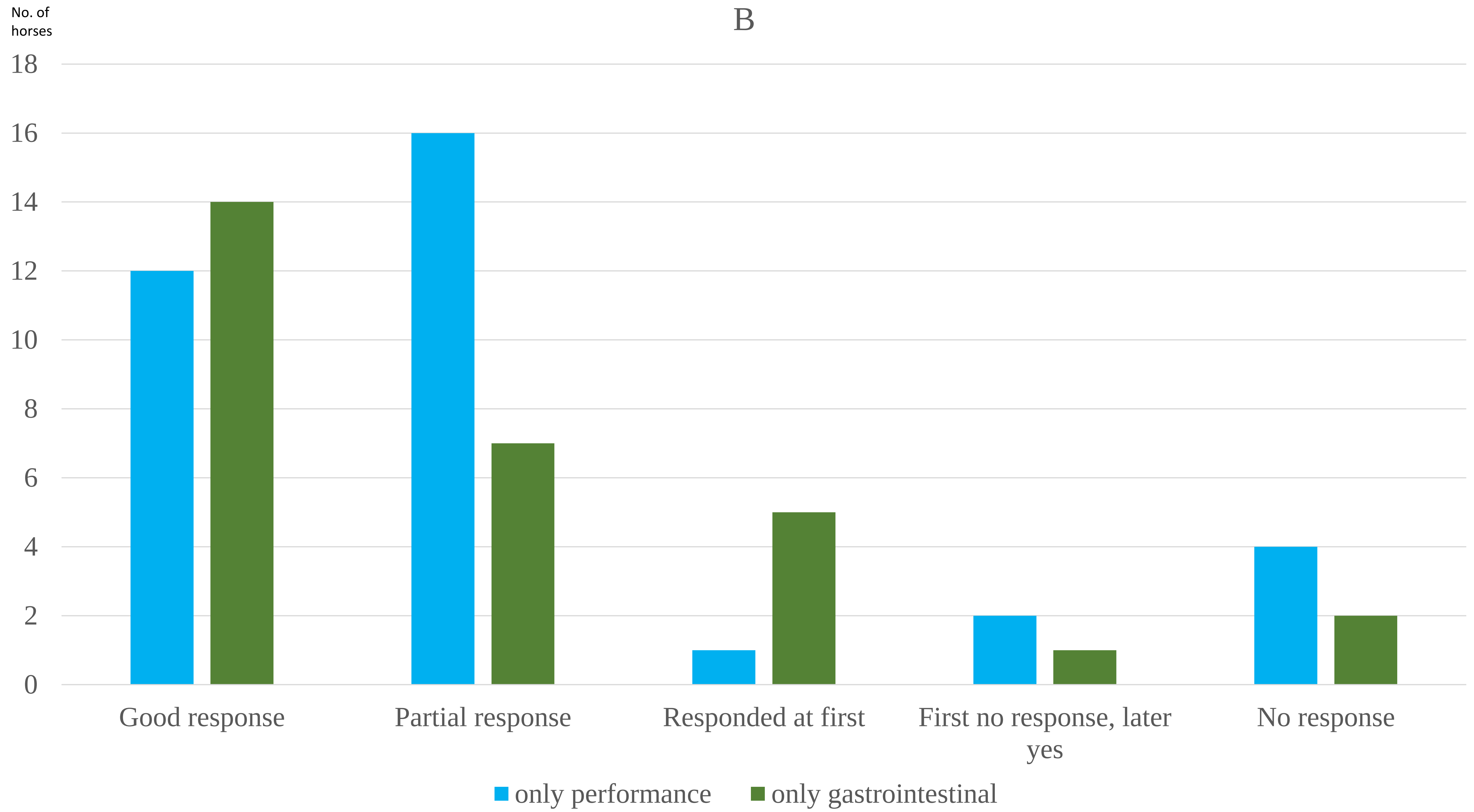
No. of horses
B
18
16
14
12
10
8
6
4
2
0
Good response
Partial response
Responded at first
First no response, later yes
No response
only performance
only gastrointestinal
